# An improved method of delivering a sclerosing agent for the treatment of malignant pleural effusion

**DOI:** 10.1186/s12885-019-5777-z

**Published:** 2019-06-24

**Authors:** Tim N. Beck, Alexander Y. Deneka, Louis Chai, Colin Kanach, Priya Johal, Nicolas J. Alvarez, Yanis Boumber, Erica A. Golemis, Glenn W. Laub

**Affiliations:** 1 Program in Molecular Therapeutics, Fox Chase Cancer Center, Philadelphia, PA 19111 USA; 2 Digestive Disease & Surgery Institute, Cleveland Clinic, 9500 Euclid Avenue, Cleveland, OH 44195 USA; 3 Department of Biochemistry, Kazan Federal University, Kazan, Russia; 4 Department of Cardiothoracic Surgery, Drexel University College of Medicine, Hahnemann University Hospital, 230 North Broad Street, Philadelphia, PA 19102 USA; 5 Department of Pathology, Drexel University College of Medicine, Philadelphia, PA 19129 USA; 6 Department of Chemical and Biological Engineering, Drexel University, Philadelphia, PA 19129 USA; 7 Department of Hematology/Oncology, Fox Chase Cancer Center, Philadelphia, PA 19111 USA

**Keywords:** Lung cancer, Metastatic cancer, Talc, Pleurodesis, Malignant pleural effusion, Mouse model, Sclerosing agent, Thermosensitive hydrogel

## Abstract

**Background:**

Malignant pleural effusion (MPE) is a devastating sequela associated with cancer. Talc pleurodesis is a common treatment strategy for MPE but has been estimated to be unsuccessful in up to 20–50% of patients. Clinical failure of talc pleurodesis is thought to be due to poor dispersion. This monograph reports the development of a foam delivery system designed to more effectively coat the pleural cavity.

**Methods:**

C57BL/6 mice were injected with Lewis lung carcinoma (LL/2) cells intrapleurally to induce MPE. The mice then received either normal saline (NS) control, foam control (F), talc slurry (TS, 2 mg/g) or talc foam (TF, 2 mg/g). Airspace volume was evaluated by CT, lungs/pleura were collected, and percent fibrosis was determined.

**Results:**

The TF group had significantly better survival than the TS group (21 vs 13.5 days, *p* < 0.0001). The average effusion volume was less in the talc groups compared to the control group (140 vs 628 μL, *p* < 0.001). TF induced significant lung fibrosis (*p* < 0.01), similar to TS. On CT, TF significantly (*p* < 0.05) reduced loss of right lung volume (by 30–40%) compared to the control group. This was not seen with TS (*p* > 0.05).

**Conclusions:**

This report describes using a novel talc foam delivery system for the treatment of MPE. In the LL/2 model, mice treated with the TF had better survival outcomes and less reduction of lung volume than mice treated with the standard of care TS. These data provide support for translational efforts to move talc foam from animal models into clinical trials.

## Background

Cancer is the second leading cause of death in the United States [1, 2], with primary lung cancer as the most common cause of cancer deaths, and death from all sources of cancer typically associated with uncontrolled metastases. Both primary lung tumors and lung metastases originating from other primary sites commonly induce a process known as malignant pleural effusion (MPE) [3]. MPE affects up to 15% of patients with cancer, and the number of patients afflicted with this complication is estimated to continue to rise as more patients experience increased overall survival with cancer, due to the availability of more effective therapies [4]. It is not entirely clear what factors determine whether MPE develops in individual patients, with more work required. Mechanistic investigations to date have associated MPE with both genomic changes (e.g., *KRAS* mutations) and immunological factors (e.g., mast cells, and immune cell-associated NF-kB signaling and TNF-alpha secretion) [5–8]. MPE is associated with worse prognosis, and patients that develop MPE have a median survival of four to 7 months [9, 10].

The pathophysiology of MPE primarily reflects vascular leakiness that results in fluid accumulation in the pleural cavity between the visceral pleura covering the lungs and the parietal pleura covering the internal aspect of the chest wall. The majority of patients suffering from MPE eventually develop dyspnea at rest [11], a significant factor in reducing their quality of life (QoL). Dyspnea develops due to compression of the lung and impaired diaphragmatic and chest wall movement [4, 9]. Symptomatic MPEs that do not respond to treatment of the underlying disease require palliative therapy directed at the pleural space. Treatment of MPE predominantly aims to relieve dyspnea and improve the patient’s overall QoL in the least invasive manner possible [12]. Typically, this is done by inducing pleurodesis (scarring) to reduce the pleural volume available for fluid accumulation. Recent work has shown that talc slurry delivered through an indwelling catheter generated successful pleurodesis in 43% of patients [13], although reported success rates vary significantly, from a low of 40% to as high as 70–80% [13–15]. In the absence of any better approaches, there is need to reduce variability and increase overall effectiveness of this technique.

This study uses a novel thermosensitive hydrogel talc foam (TF) designed to more effectively coat the pleural cavity and thereby provide more durable and more reliable chemical pleurodesis. We use an immunocompetent mouse model of MPE [5, 6, 16, 17] to evaluate whether a TF delivery system is an improved method of delivering talc for the treatment of MPE. The primary aim of this study is to investigate whether TF delivery to the pleural cavity is effective, and whether TF is a viable treatment option for MPE.

## Methods

### Cell culture

Lewis lung carcinoma (LL/2) cells transduced with LV-Fluc-P2A-Puro (identifier: LL/2-Fluc-Puro) were purchased in 2018 directly from Imanis Life Sciences (Rochester, MN, USA), who authenticate the cell lines prior to shipment. The cell line was tested for mouse pathogens prior to use and were negative. LL/2 cells are mouse cells and do not require ethical approval. Cells were cultured at 37 °C in 5% CO_2_ using DMEM supplemented with 10% fetal bovine serum, 1x Penicillin/Streptomycin and 2 μg/mL puromycin.

### Foam, talc foam (TF) and talc slurry (TS)

**Fig. 1:**
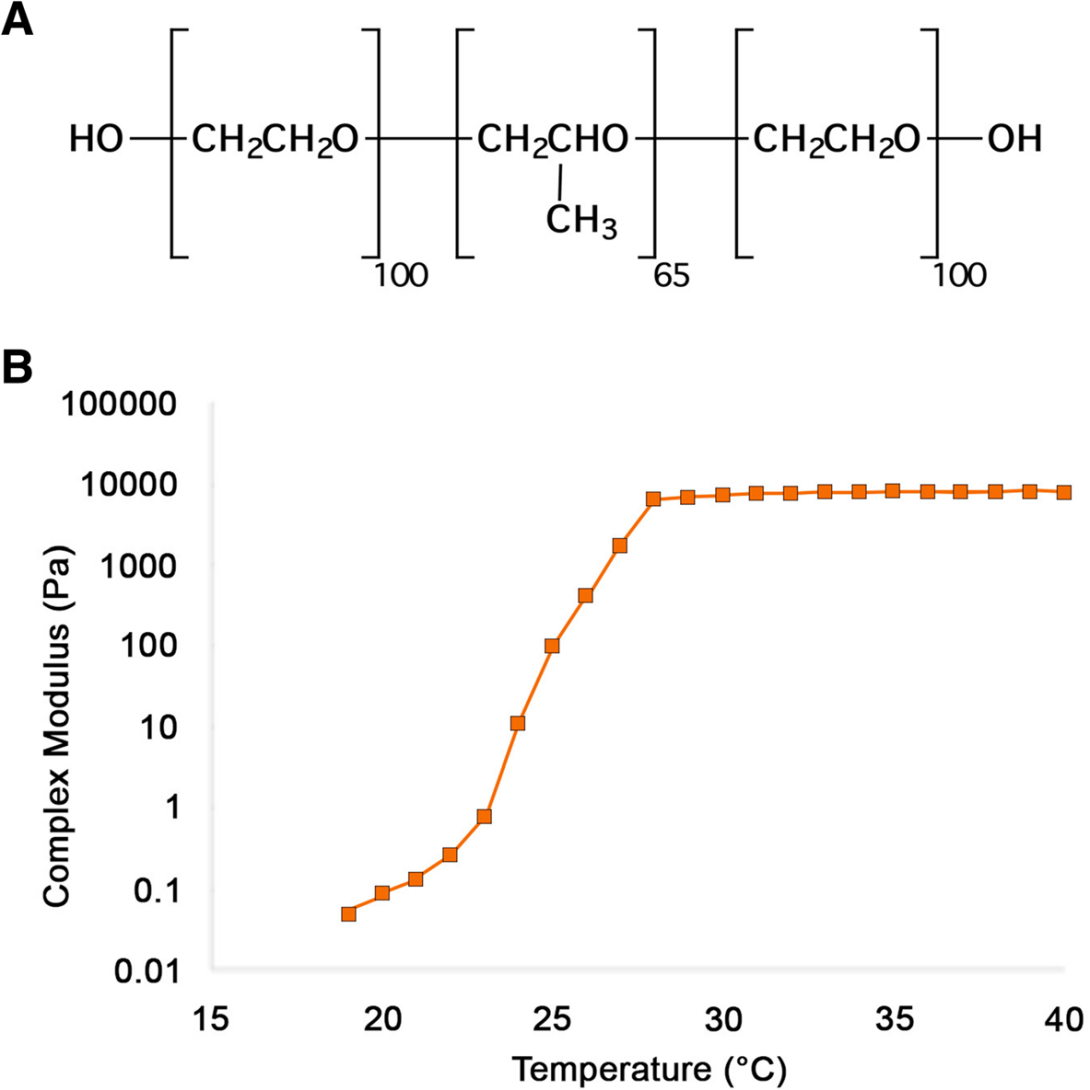
Triblock copolymer hydrogel for delivery of talc. **a**. Molecular structure of the polymer matrix. PEO = polyethylene oxide; PPO = polypropylene oxide. **b**. Rheological studies of a triblock copolymer demonstrating reverse temperature dependent viscosity. As the temperature increases, the viscosity increases several orders of magnitude. Complex modulus in Pascals (Pa)

The foam delivery system is comprised of a triblock copolymer [18] hydrogel (Fig. 1a) in a saline solution that exhibits a reverse thermosensitive viscosity profile. A liquid at room temperature, the hydrogel undergoes a sol-gel transition at temperatures greater than ~ 25 °C (Fig. 1b). When foamed by agitating the cooled liquid hydrogel with air, the resultant foam is a thin liquid. At physiological temperatures, the foam rapidly collapses and forms a viscous, sticky gel.

The cooled liquid was loaded into a 10 cc syringe and aerated using a Disofix three-way stopcock (B. Braun Medical Inc., Bethlehem, PA) and a second 10 cc syringe filled with air at a 1:1 ratio of air and liquid. Foam was generated by mixing the air and gel through the three-way stopcock until the desired consistency of foam was obtained. The foam was freshly prepared shortly before injection and kept on ice until immediately before each injection.

For the talc foam, the above-mentioned cooled liquid was combined with talc (powder, 10 μm, Millipore-Sigma) within a 10 cc syringe, to achieve a concentration of 2 mg talc per gram of mouse body weight (2 mg/g). The mixture was aerated using a 1:1 ratio of air and the gel-talc mixture, to generate TF.

TS was prepared as previously described [19, 20] to a final concentration of 2 mg/g. In brief, talc was mixed with sterile saline using two 10 cc syringes connect via a three-way stopcock. TS was freshly prepared shortly before injection and kept on ice until right before each injection.

### Rheology

Rheological studies of the triblock copolymer hydrogel (Fig. 1b) were performed on a DHR-3 rheometer, TA Instruments (Calumet, MI) using parallel plates, 40 mm in diameter, with a gap of 1 mm. A Peltier system was used to perform temperature ramps by heating the plates and all data were collected in the linear regime of the amplitude. A thin layer of silicone oil was added surrounding the plates to prevent evaporation during the experiment. A heating rate of 1.45 °C/min was used. The complex modulus, G*, storage modulus, G’, and loss modulus G”, were measured for each of the formulations. The complex modulus is related to the storage modulus and loss modulus by equation 1 (Eq. 1).

1 $$ {G}^{\ast }=\sqrt{G^{\hbox{'}2}+}\kern0.5em {G}^{"2} $$

### Computer tomographic (CT) imaging and quantitative analysis

Mice were anesthetized with 2% isoflurane in O_2_ for induction and were then maintained in 0.5% isoflurane during the scan. Scanning was performed with a Sofie Biosciences CT/PET machine (G8 PET/CT, PerkinElmer/Sofie Biosciences, Culver City, CA) using the standard protocol specified by the manufacturer. To calculate airspace volume, VivoQuant software (inviCRO, Boston, MA) was used. To identify the total area of air-filled lung, areas with minimal contrast within a specific range, were automatically highlighted using the Connected Thresholding function. This setting was kept consistent for all analyzed mice. The airspace volume was calculated automatically in mm^3^. Trachea and other artifacts were excluded manually. Mice were scanned immediately after the injection of tumor cells and again at the end of the experiment. The pre-injection scan for each mouse served as the reference scan used to calculate the remaining lung volume for each treatment.

### Murine model

All animal care and experimental procedures were prospectively approved by the Fox Chase Cancer Center Institutional Animal Care and Use Committee (IACUC). The Fox Chase Cancer Center staff tended to the mice daily and for the duration of the experiment. 6–8-week-old male and female C57BL/6 mice (obtained from The Jackson Laboratory, Bar Harbor, ME) were acclimatized for 1 week before use.

To first assess TF as a sclerosing agent, mice without tumors were randomly assigned into one of the following groups: control (foam or saline), TF, or TS. For the TS group or for TF (prepared as described above), the volume injected was adjusted based on body weight, to achieve a final injected dose of 2 mg talc per gram mouse body weight. The volume for foam or saline was calculated to match the volumes used for the talc treatment group. Talc mixtures (TF or TS) were then injected intrapleurally into the right pleural cavity. Mice were anesthetized with isoflurane and the right chest was cleaned with an alcohol solution. A 23-gauge needle attached to a 1 mL syringe was introduced into the right chest cavity at 1 cm lateral to the right parasternal line, as previously described [17]. The mice were euthanized 7 days post-treatment, and the chest cavity was evaluated for fibrosis as described below.

To generate MPE, intrapleural injection of LL/2 cells was performed. Mice were anesthetized with 1–3% isoflurane gas and the right chest was cleaned with an alcohol solution. A 23-gauge needle attached to a 1 mL syringe containing 1.5 × 10^5^ of LL/2 cells in sterile PBS was introduced into the right chest cavity at 1 cm lateral to the right parasternal line. The needle was slowly advanced until it reached the pleural space. The cell suspension was then carefully injected. All mice were monitored until completely recovered from the procedure. Four days after the injection of LL/2 cells, all mice were randomly assigned into one of the following groups: control (foam or saline), TF, or TS, each of which were injected as described in the previous paragraph.

For effusion and survival analyses, mice were injected with LL/2 cells as described above, then underwent CT imaging 1 day and 14 days after the injection of cells, to assess lung volume status. Four days after the injection of cells, all mice were randomly assigned to receive intrapleural control foam/saline, TF, or TS, at the appropriate concentration and volume as noted above. The mice were euthanized (CO_2_ inhalation) according to the approved IACUC protocol upon showing signs of poor health, presentation with signs of pain or distress, or if they experienced a rapid loss of weight (> 20% of body weight over 7 days).

### Histopathological evaluation

Immediately following euthanasia, the abdominal wall of the mice was opened, and the viscera were retracted to visualize the diaphragm, which was punctured with a 23-gauge needle to aspirate pleural fluid. The pleural fluid volume was measured. The thorax was dissected and removed *en bloc*. All lungs were imaged and collected for histology. Lungs were fixed in 10% phosphate-buffered formaldehyde for 24–48 h, dehydrated by incubation in ethanol followed by xylene (70% ethanol, 3 h; 95% ethanol, 2 h; 100% ethanol, 2 h; ethanol-xylene, 1 h; xylene, 3 h) then embedded in paraffin. 5 μm thick slices were cut, mounted on slides and stained with trichrome. Stained slides were scanned with Vectra 2.3 Automated Quantitative Pathology Imaging System (Perkin Elmer, Waltham, MA). Tissue segmentation (% fibrosis) was determined by automated quantitative analysis using InForm software (PerkinElmer Inc., Waltham, MA).

### Statistics

For all experiments indicated, *p* values were calculated using one-way ANOVA (GraphPad Prism version 6.00 for Mac; GraphPad Software) or Student’s t test as specified. Survival curves were generated using the Kaplan and Meier method and tested for significance using log-rank tests.

In justification of animal number in experimental cohorts, we would set the Type I error rate to 5%/3 = 1.67% Type I error (2-sided). In this case with a relatively small number of multiple comparisons, the Bonferroni correction sets the *p*-value to a level that is more generous than using a 1% False Discovery Rate (FDR). With 10 animals per cohort, we would have 85% power to detect a standardized effect of 1.7 standard deviation units. We used generalized linear models (GLMs) for analysis, assuming normal distribution and identity link.

## Results

### Talc foam is an effective sclerosing agent

**Fig. 2:**
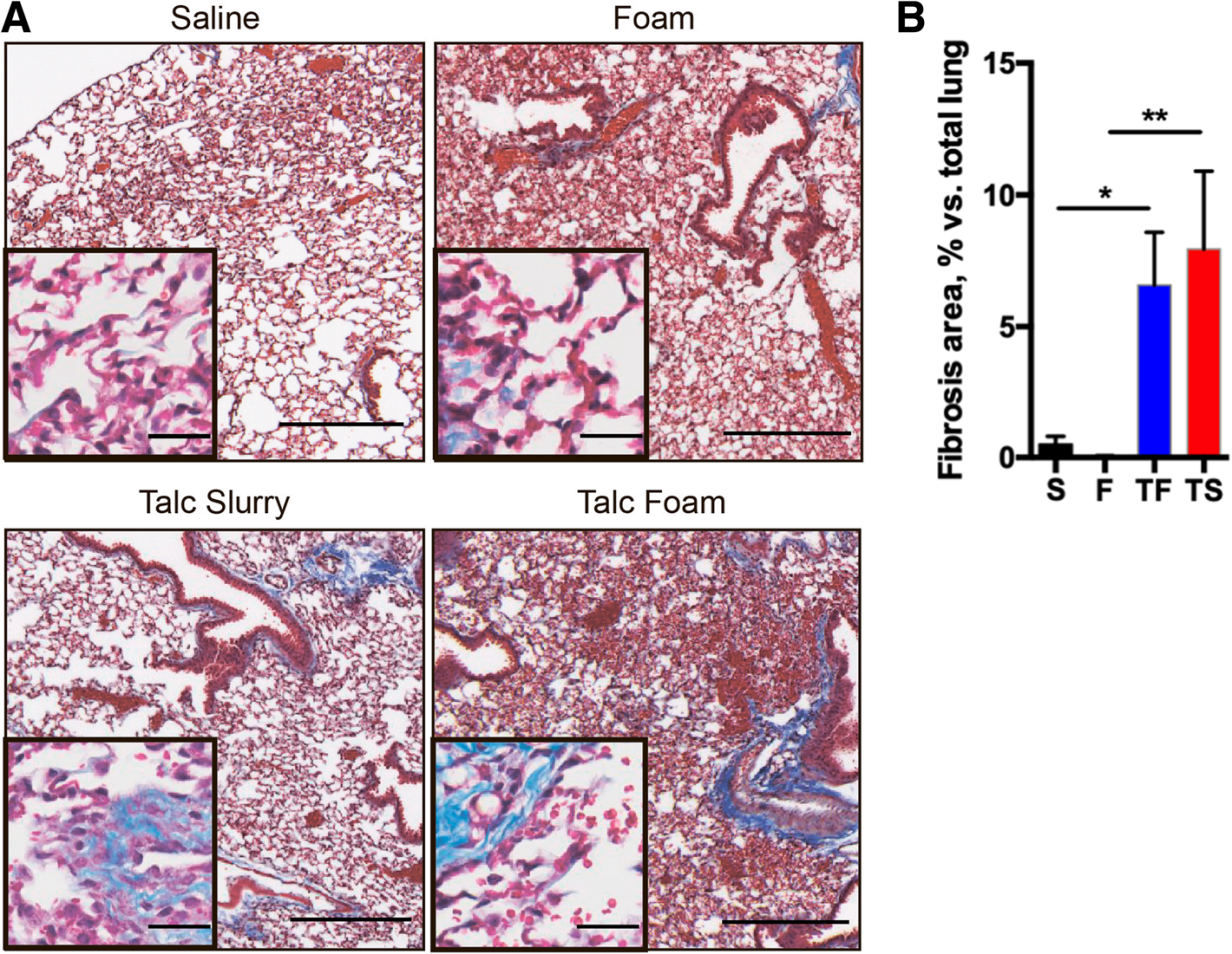
Histology of pleura and lung parenchyma of mice that received intrapleural injection of saline (S), foam (F), talc foam (TF) or talc slurry (TS). **a**. Representative images of hematoxylin-eosin and trichrome stained tissue samples. Trichrome stains fibrotic tissue component and appears in blue color. Large image: magnification 4x, scale bar, 300 μm; inset: 20x, scale bar: 30 μm. **b**. Quantification of fibrotic lung and pleural tissue for each treatment group. *, *p* < 0.05; **, *p* < 0.01. Number of animals per group = 3 for F and NS, 8 for TS and 11 for TF

We evaluated the sclerosing potential of talc foam (TF; Fig. 1), which transitions from liquid to gel at temperatures above 29 °C, in comparison with talc slurry (TS), foam (F), or saline (S). Analysis of trichrome stained lung tissue from mice after intrapleural injection with S, F, TS, or TF revealed significant fibrosis in the lungs of mice receiving TS or TF (Fig. 2a). Fibrosis quantified by Vectra from trichrome-positive tissue ranged from 0.19 to 21.4% of the total lung volume across all groups. Fibrosis averaged 6–7% of the lung in mice receiving TF, versus 8% in those receiving TS; a statistically insignificant difference. Importantly, the percent fibrosis was significantly higher in the TF (*p* < 0.05) and TS (*p* < 0.01) groups compared to the lung fibrosis detected for S- or F- treated mice (Fig. 2b).

### Talc foam reduces loss of lung volume

**Fig. 3:**
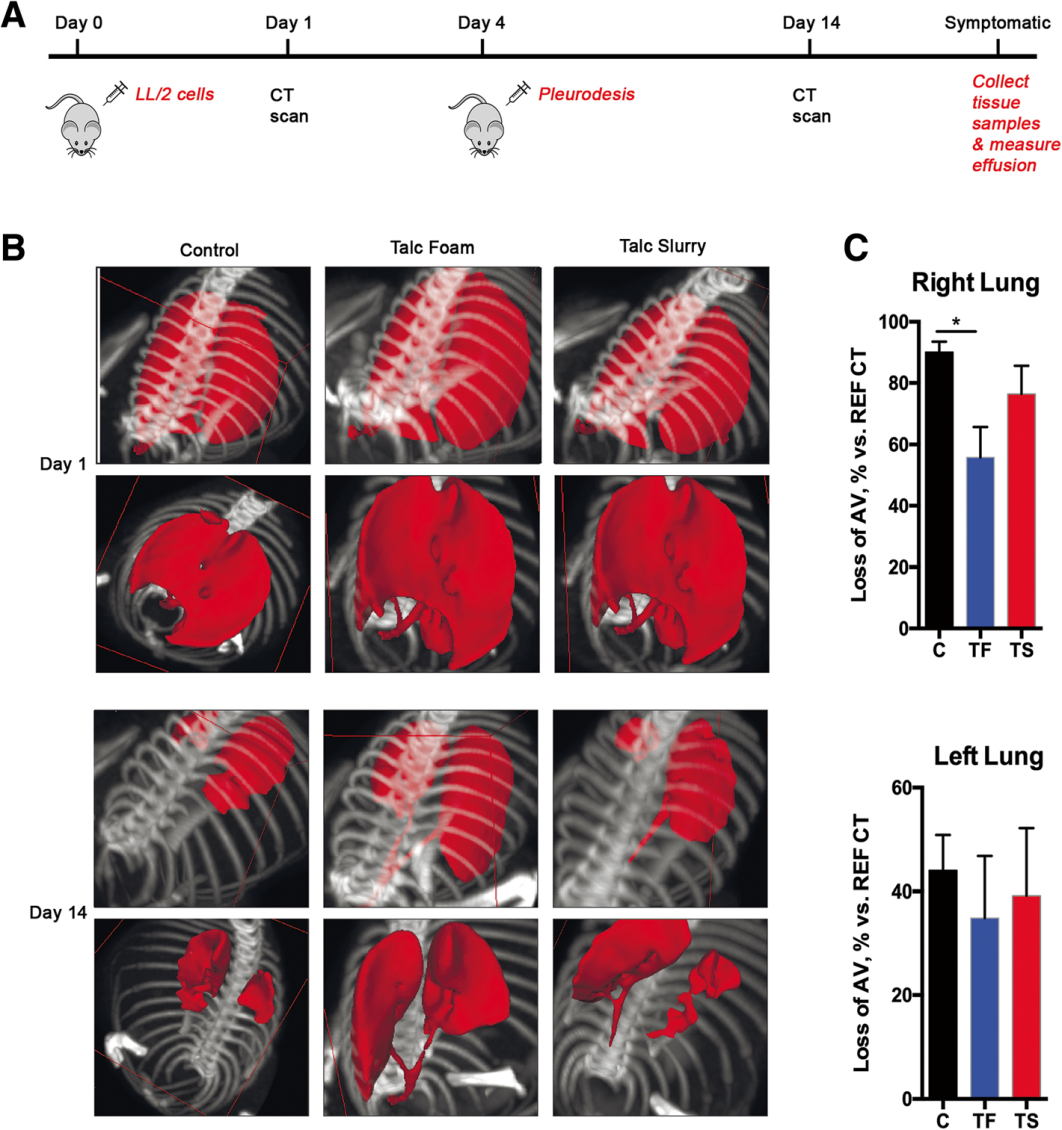
Evaluation of loss of air volume after treatment with control treatment, talc foam (TF) or talc slurry (TS). **a**. Study design. **b**. Representative CT images for the three treatment groups (control – C – foam and saline; talc slurry – TS; talc foam – TF) at two different time points. Detected air volume (AV) is shown in red. **c**. Average loss of air volume based on CT scanning at day 14, compared to day 1 (considered 100%) for each mouse. *, *p* < 0.05. Number of animals per group = 6 for C, 6 for TS and 8 for TF

As a model to evaluate TF effectiveness for MPE, mice were injected with LL/2 cells into the right pleural cavity as described in the methods section. All mice underwent CT imaging 1 day after the injection, without any significant difference noticed between mice (data not shown). The mice were then randomly selected to receive control S or F, TF, or TS, in the right pleural cavity, which was administered 4 days after the injection of LL/2 cells, followed by re-imaging at day 10 (Fig. 3a). CT imaging of the lungs of euthanized mice indicated that TF talc foam effectively reduced loss of lung volume (Fig. 3b). Quantification of CT data demonstrated that TF significantly (*p* < 0.05) reduced loss of right lung volume (by 30–40%) compared to the loss of lung volume in the control group. This was not seen in the TS group (*p* > 0.05; Fig. 3c). No differences between the left lung volume was observed for the three groups (Fig. 3c).

### Talc foam reduces MPE volume and provides superior survival compared to talc slurry

**Fig. 4:**
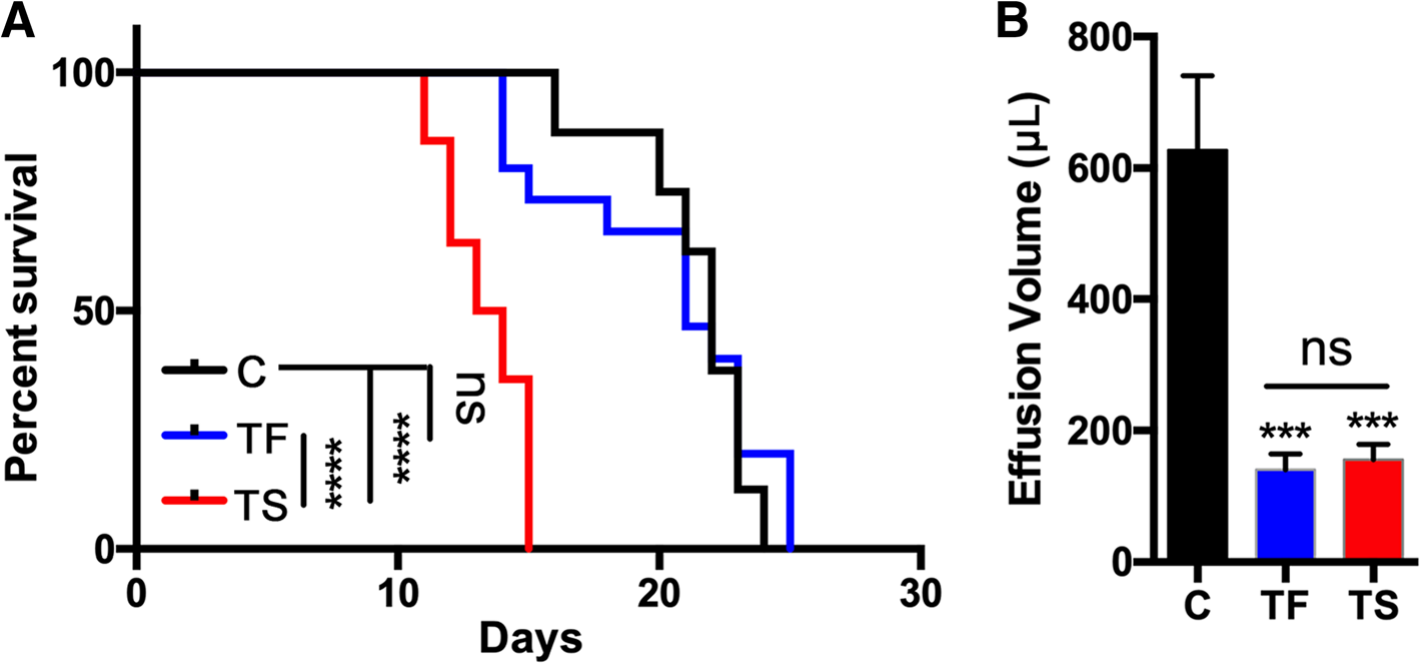
Talc foam reduces MPE without adversely impacting survival. **a**. Kaplan-Meier survival curve of mice treated with control (foam or saline), TF, or TS. Number of animals per group = 8 for control, 15 for TF and 14 for TS. **b**. Effusion volume (μL) for each treatment group. Not significant (ns), *p* > 0.05; ***, *p* < 0.001; ****, *p* < 0.0001

To evaluate if TF influenced survival outcomes, mice were injected with LL/2 cells in the right pleural space as described above, and randomly assigned to treatment with S, F, TF, or TS. They were then observed until signs of distress appeared, and euthanized. The TF group and both the F and S control groups had similar median survival durations (21 days and 22 days, respectively), without a statistically significant difference. However, mice treated with the standard of care TS, compared to the other two groups, had significantly reduced overall survival of only 13.5 days (*p* < 0.0001; Fig. 4a).

Following euthanasia, the effusion volume was directly measured for each mouse. Mice in the control groups had an average effusion volume of 627.5 μL. In contrast, the average effusion volumes for TS treated mice and TF treated mice were 155.7 μl and 140.0 μl, respectively (Fig. 4b). The difference in effusion volume between both the TS- and TF- treated cohorts versus the control group was statistically significant (*p* < 0.001). No significant difference was detected between these two treatment groups.

## Discussion

Managing MPEs presents one of the greatest treatment challenges of cancer-associated complications. In the US alone, an estimated 150,000 patients develop MPE annually [21]. Talc slurry pleurodesis can provide significant relief to patients with MPE; however, the incomplete effectiveness of chemical pleurodesis [13–15] is not ideal, while alternatives such as indwelling pleural catheters can interfere with a patient’s QoL due to infection, cellulitis and catheter tract metastasis [22, 23]. The talc foam described in this monograph presents a novel means of delivering a sclerosing agent more effectively to the pleural space of mice and could potentially provide improved outcomes for patients afflicted with malignant pleural effusions.

In this study, a previously described mouse model of MPE [5, 6, 16, 17] was used to test the efficacy of a novel foam delivery system designed to improve the dispersal of agents injected into the pleural space. Talc was successfully delivered into the pleural space and established fibrotic changes detected by trichrome staining, similar to fibrosis induced using the standard of care talc slurry. Intriguingly, talc foam significantly prevented loss of air volume, compared to the control treatment, which was not the case for the talc slurry. This difference is likely due to improved dispersion of the foam within the pleural cavity. Talc foam did not adversely affect survival compared to the control group, and demonstrated significantly better survival compared to conventional pleurodesis. The reduction in survival between the different treatment groups is again likely a result of the poor distribution of the talc slurry, which may have caused cardiovascular compromise due to more focused pressure on the heart. Further studies are needed to explore these observed survival differences. However, it is unlikely that these findings would translate to the clinic, given that chemical pleurodesis has not been shown to negatively impact survival, but rather improves quality of life. Additionally, talc foam reduced effusion volumes substantially. The amount of pleural effusion was not significantly different between the TS and the TF groups (Fig. 4), making it less likely that the effusion volume was a significant confounding factor on the loss of right lung volume. The perhaps most critical aspect of treating MPE is effective reduction of the effusion, given that dyspnea is the most common symptom of MPE [24]. These results suggest that foam delivery of talc is safe and effective and proposes that TF would perform as well if not better in the clinic than the standard of care TS.

The novel foam delivery system described in this study presents an entirely new potential platform for efficient intrapleural treatment delivery beyond talc pleurodesis that may also be helpful in controlling MPE. For example, recent work has demonstrated that anti-EGFR and anti-VEGF drugs administered intrapleurally can reduce effusion volume and inflammatory mediators in pleural fluid and thereby decrease morbidity due to MPE [24]. It is also known that *KRAS* mutation-driven CCL2 and HSP90/IKKα/IKKβ regulated IL-1β signaling clearly plays a role in the development of MPE [5, 6, 8]. An interesting next step would be evaluation of intrapleural delivery by foam delivery of therapeutic agents such as anti-EGFR and anti-VEGF drugs, HSP90 inhibitors [25, 26] and bortezomib [6] to inhibit HSP90/IKKα/IKKβ activity. Therefore, efficient delivery and intrapleural dispersion of a variety of therapeutic agents could significantly improve how MPEs are managed. Future studies will focus on using foam delivery to achieve favorable dispersion at therapeutic concentrations of different sclerosing and therapeutic agents within the pleural space in animal models as well as in clinical studies. This approach has the potential to alter the clinical course for many cancer patients and improve their quality of life.

## Conclusions

Reverse thermosensitive hydrogel foam delivery of talc is an effective way of treating MPE in an established mouse model [5, 6, 16, 17]. MPE is a devastating sequela of cancer and additional therapeutic approaches are desperately needed. The findings discussed in this report provide the foundation for future studies of talc foam in more clinically relevant settings as well as for future explorations of intrapleural delivery of different therapeutic agents. The role of pleurodesis and the need for additional sclerotic agents may increase further as immunotherapies continue to improve overall and progression free survival of cancer patients [27–30]. Our findings lay the foundation for advanced translational clinical studies of talc foam, to further investigate the potential this treatment holds for improving the QoL of patients suffering from MPE.

## Data Availability

All data is included as part of the manuscript or as part of the supplemental materials section.

## Abbreviations

ANOVA: Analysis of variance
AV: Air volume
CCL-2: Chemokine (C-C motif) ligand 2
CT: Computer tomography
EGFR: Epidermal growth factor receptor
F: Foam control
FDR: False discovery rate
GLMs: Generalized linear models
HSP90: Heat shock protein 90
IACUC: Institutional Animal Care and Use Committee
IKKα: Iκb kinase α
IKKβ: IκB kinase β
IL-1β: Interleukin 1 beta
KRAS: Kirsten Rat Sarcoma Viral Proto-Oncogene
LL/2: Lewis lung carcinoma
MPE: Malignant pleural effusion
NF-kB: Nuclear factor kappa-light-chain-enhancer of activated B cells
NS: Normal saline
PEO: Polyethylene oxide
PET/CT: Positron emission tomography/computer tomography
PPO: Polypropylene oxide
QoL: Quality of life
TF: Talc foam
TNFα: Tumor necrosis factor-α
TS: Talc slurry
VGEF: Vascular endothelial growth factor
